# Beating the heart failure odds: long-term survival after myocardial ischemia in juvenile rainbow trout

**DOI:** 10.1152/ajpregu.00005.2024

**Published:** 2024-02-26

**Authors:** Lucas A. Zena, Andreas T. Ekström, Daniel Morgenroth, Tristan McArley, Michael Axelsson, Henrik Sundh, Anders Palmquist, Ida B. Johansen, Albin Gräns, Erik Sandblom

**Affiliations:** ^1^Department of Biological and Environmental Sciences, University of Gothenburg, Gothenburg, Sweden; ^2^Department of Applied Animal Science and Welfare, Swedish University of Agricultural Sciences, Gothenburg, Sweden; ^3^Department of Biomaterials, University of Gothenburg, Gothenburg, Sweden; ^4^Department of Preclinical Sciences and Pathology, Faculty of Veterinary Medicine, Norwegian University of Life Sciences, Ås, Norway

**Keywords:** cardiac biomarkers, electrocardiogram, myocardial ischemia, myocardial scarring, revascularization

## Abstract

Salmonid fish include some of the most valued cultured fish species worldwide. Unlike most other fish, the hearts of salmonids, including Atlantic salmon and rainbow trout, have a well-developed coronary circulation. Consequently, their hearts' reliance on oxygenation through coronary arteries leaves them prone to coronary lesions, believed to precipitate myocardial ischemia. Here, we mimicked such coronary lesions by subjecting groups of juvenile rainbow trout to coronary ligation, assessing histomorphological myocardial changes associated with ischemia and scarring in the context of cardiac arrhythmias using electrocardiography (ECG). Notable ECG changes resembling myocardial ischemia-like ECG in humans, such as atrioventricular blocks and abnormal ventricular depolarization (prolonged and fragmented QRS complex), as well as repolarization (long QT interval) patterns, were observed during the acute phase of myocardial ischemia. A remarkable 100% survival rate was observed among juvenile trout subjected to coronary ligation after 24 wk. Recovery from coronary ligation occurred through adaptive ventricular remodeling, coupled with a fast cardiac revascularization response. These findings carry significant implications for understanding the mechanisms governing cardiac health in salmonid fish, a family particularly susceptible to cardiac diseases. Furthermore, our results provide valuable insights into comparative studies on the evolution, pathophysiology, and ontogeny of vertebrate cardiac repair and restoration.

**NEW & NOTEWORTHY** Juvenile rainbow trout exhibit a remarkable capacity to recover from cardiac injury caused by myocardial ischemia. Recovery from cardiac damage occurs through adaptive ventricular remodeling, coupled with a rapid cardiac revascularization response. These findings carry significant implications for understanding the mechanisms governing cardiac health within salmonid fishes, which are particularly susceptible to cardiac diseases.

## INTRODUCTION

The heart is a highly aerobic organ that depends on a constant supply of oxygen for maintaining viability and function. A prolonged reduction or complete blockage of blood flow of the coronary vessels, which supplies oxygen-rich blood to the heart, can result in myocardial ischemia and infarction, commonly referred to as a heart attack (1). In mammals, cardiomyocyte death following myocardial infarction triggers an intense inflammatory response that ultimately results in the replacement of damaged tissue with a fibrotic scar (2). Myocardial infarction can lead to structural and electrophysiological cardiac remodeling that is often associated with various types of arrhythmias (i.e., abnormal heart rhythms). In addition, infarction may disturb and alter the sympathetic and parasympathetic autonomic innervation of the heart, potentially exacerbating the development of arrhythmias (3). Despite advances in the diagnosis and treatment of myocardial infarction, mortality remains high, with 7–20% of deaths occurring within 1 yr (4). Sudden death is most often attributed to various arrhythmias, including heart blocks, ventricular tachycardia, and fibrillation (5, 6).

Although a heart attack results in permanent damage to the heart in mammals, some studied fish species can restore cardiac structure and function after heart injury (7, 8). For instance, myocardial injury, repair, and remodeling have been extensively studied in zebrafish, which is an established model for comparative studies of the evolution, pathophysiology, and ontogeny of vertebrate heart regeneration (9, 10). It is worth noting that cardiac morphology, anatomy, and associated coronary circulation exhibit significant variability across different fish species. In the zebrafish heart, as in the hearts of many other teleosts, the contribution of the coronary circulation providing oxygen-rich blood to the myocardium seems to be small. This is because the zebrafish heart primarily consists of the spongy myocardium, with the compact layer comprising maximum four cell layers (11). The spongy myocardial tissue predominantly relies on the scarce amounts of oxygen present in venous blood returning to the heart from body tissues, which diffuses into the spongy myocardium layer [i.e., the luminal supply (12–14)]. Yet, the zebrafish stands out as a model for studying cardiac remodeling following injury-induced myocardial ischemia. Commonly used methods for this purpose include ventricular apex amputation, cryoinjury, and cardiomyocyte genetic ablation (9). Even so, these methods may not accurately replicate an actual event of myocardial ischemia and can even interfere with the overall integrity and structure of the heart, including the inner spongy myocardium (15).

Salmonids, such as rainbow trout (*Oncorhynchus mykiss*) and Atlantic salmon (*Salmo salar*), have a more developed ventricular compaction comprising 20–50% of the total ventricular mass (8, 16–20), and the cardiac cells within this tissue rely on coronary circulation for delivery of oxygen-rich blood (12). While offering increased cardiac force generation, an extensive compact myocardium perfused with coronary arteries renders the salmonid heart vulnerable to diseases and pathological alterations of the coronary vessels. For instance, both wild and farmed salmonids are highly susceptible to developing coronary lesions caused by vessel wall neointimal vascular smooth muscle cell proliferation that narrows the coronary artery lumen [i.e., coronary arteriosclerosis; (21)]. This condition may reduce blood flow to the heart muscle, leading to myocardial ischemia (21, 22).

The electrocardiogram (ECG), which measures the electrical activity of the heart, is a comparably simple and cost-effective clinical diagnostic tool to detect various cardiac abnormalities and diseases, especially arrhythmias and acute myocardial infarction (23). Consequently, early detection of cardiac abnormalities through the electrocardiogram could potentially help foresee the morbidity and mortality of farmed salmonids (24). The ECG of fish is similar to that of humans (25) and comprises a P wave representing atrial depolarization, the QRS complex representing ventricle depolarization, and the T wave representing ventricular relaxation and repolarization. We have recently shown that acute coronary occlusion in anesthetized rainbow trout evokes human-like ECG characteristics of heart attack, including various degrees of atrioventricular blocks, delayed ventricular depolarization (i.e., prolonged QRS duration), reduced QRS amplitude, and abnormalities in the ST-segment, which is the interval between ventricular depolarization and repolarization (24). The latter is considered the most acute indicator of severe coronary artery disease in humans (1), thus demonstrating the usefulness of the ECG in detecting heart diseases in fish.

The potential implications of coronary arteriosclerosis on cardiac function in salmonid fish are supported by laboratory experiments in which acute (short-term, 1 to 7 days) blockade (by surgical ligation) of the main coronary artery results in compromised swimming performance, aerobic metabolic performance as well as reduced tolerance to warming and hypoxia (20, 26–31). Yet, the long-term consequences of myocardial ischemia due to coronary obstruction on cardiac function and morphology are still poorly understood. Furthermore, experimental coronary artery occlusion in trout results in significant mortality in large fish within 10 days of the onset of ischemia [e.g., approximate body mass of 900 g; (24)], while juvenile fish (approximately 250 g) develop significant short-term (i.e., 3-day) impaired cardiac function, such as elevated resting heart rate and reduced heart rate variability, along with reduced heart rate scope (maximum-resting heart rate) (8), but typically survive and eventually are able to restore normal cardiac function (i.e., in 1 to 2 mo). Yet, surviving rainbow trout develop an extensive myocardial scarring in the midmyocardial compact layer (8). Myocardial scarring after myocardial infarction may create regions of slowed conduction and re-entrant circuits supporting the appearance of ventricular arrhythmias (5). Indeed, zebrafish exhibit abnormal repolarization (long QT interval), as well as ST-segment depression, coinciding with scar formation following cardiac cryoinjury or resection (15, 32). In contrast, methods such as occlusion of the main coronary artery in rainbow trout result in scar tissue formation limited to the median portion of the compact myocardium (i.e., intramural scar), while the surrounding compact and spongy cardiac muscle remains unaffected (8). The lack of scarring in other portions of the compact is somewhat surprising given that the main coronary artery serves as the conduit for oxygenated blood to reach the whole compact myocardium layer in these species (22). However, the reason for the unusual midmyocardium fibrous layer formation, its contribution to the sustained long-term survival in juvenile rainbow trout, and its implications for the electrical activity of the trout heart remain unknown (8). Nonetheless, apart from the presence of the midmyocardium fibrous layer, the seemingly healthy surrounding compact myocardial tissue in the long term may underlie the re-establishment of a coronary blood supply rich in oxygen, a feat that could only be accomplished through coronary vessel regrowth (8).

In the present study, we subjected groups of juvenile rainbow trout to coronary artery ligation and allowed the fish to recover for 3 days (i.e., short-term recovery group) or for up to 173 days (i.e., long-term recovery group). The purpose was to test the hypothesis that impairments in cardiac function following coronary ligation are explained by underlying morphohistological changes occurring from initial myocardial ischemia, followed by cardiac fibrosis occurring as a result of cardiac injury. For this, we assessed in vivo cardiac functions such as heart rate, rhythm, and autonomic control using high-resolution ECG recording. We also analyzed ventricular morphological characteristics to examine the prevalence and histological features of acute myocardial ischemia and cardiac changes and remodeling following initial infarction and consequent fibrosis deposition. Finally, we assessed the occurrence of myocardial revascularization to determine whether coronary regrowth underlies long-term survival previously observed after coronary ligation in juvenile trout (8).

## MATERIALS AND METHODS

### Experimental Animals

Juvenile rainbow trout *Oncorhynchus mykiss* (Walbaum, 1792) of mixed sexes (see Table 1 for biometrics) were obtained from a local fish farm (Vänneåns Fiskodling AB, Sweden) and transported to the University of Gothenburg in April 2021. Before the experiments started, the fish were kept in holding tanks with recirculating aerated freshwater (10°C) and a photoperiod of 12:12 h light:dark for at least 4 wk. The fish were fed three times a week with commercial fish pellets throughout the experiment (from May to December 2021). Ethical permit no. 5.8.18-10907/2020 issued from the regional animal ethics committee in Gothenburg covered all experimental procedures.

**Table 1.** Body size metrics and results from the linear model for body mass, standard length, and condition factor in short-term (3 days) and long-term (114 and 170 days) recovery in nonanesthetized sham-operated and coronary-ligated rainbow trout (Oncorhynchus mykiss)

	Body Mass, g	Short Term vs. Long Term	Sham vs. Ligated	Interaction
Short term		***F*_(1,44)_ = 100.86; *P* < 0.001**	*F*_(1,44)_ = 1.41; *P* = 0.24	*F*_(1,44)_ = 0.95; *P* = 0.33
Sham operated, *n* = 12	445.0 ± 20.5			
Coronary ligated, *n* = 13	501.9 ± 27.0			
Long term				
Sham operated, *n* = 9	816.3 ± 37.0			
Coronary ligated, *n* = 14	837.0 ± 53.5			

	Standard Length, cm	Short Term vs. Long Term	Sham vs. Ligated	Interaction
Short term		***F*_(1,44)_ = 108.60; *P* < 0.001**	*F*_(1,44)_ = 0.55; *P* = 0.46	*F*_(1,44)_ = 1.60; *P* = 0.21
Sham operated, *n* = 12	31.3 ± 0.3			
Coronary ligated, *n* = 13	32.3 ± 0.4			
Long term				
Sham operated, *n* = 9	37.7 ± 0.5			
Coronary ligated, *n* = 14	37.4 ± 0.7			

	Condition Factor	Short Term vs. Long Term	Sham vs. Ligated	Interaction
Short term		***F*_(1,44)_ = 5.73; *P* = 0.02**	*F*_(1,44)_ = 1.45; *P* = 0.23	*F*_(1,44)_ = 0.13; *P* = 0.72
Sham operated, *n* = 12	1.45 ± 0.05			
Coronary ligated, *n* = 13	1.48 ± 0.03			
Long term				
Sham operated, *n* = 9	1.52 ± 0.04			
Coronary ligated, *n* = 14	1.57 ± 0.02			

The condition factor of the fish was calculated as: (100 × body mass)/standard length^3^. Statistically significant (*P* ≤ 0.05) findings are in bold.

### Surgical Procedures and Experimental Protocols

Fish were initially anesthetized in freshwater (10°C) containing MS-222 (tricaine methanesulfonate, 150 mg·L^−1^) buffered with NaHCO_3_ (300 mg·L^−1^) and then positioned on their left lateral side on a surgery table covered with wet foam. To maintain the fish anesthetized throughout the surgical procedures, 10°C water containing MS-222 at the concentration of 75 mg·L^−1^ buffered with 150 mg·L^−1^ NaHCO_3_ was continuously irrigated over the gills. An incision was made in the isthmus to expose the coronary artery (19). In one experimental group, the coronary artery was completely blocked by tying a 6-0 silk suture around the vessel (coronary-ligated group). A second group was treated identically except that the coronary artery was not ligated (sham-operated group). ECG electrodes were prepared by soldering 23-gauge hypodermic needles, with the beveled edge blunted, onto three insulated stainless-steel wires. Two ECG electrodes were implanted subcutaneously close to the pectoral fins on each side of the heart and sutured in place. The negative electrode was implanted close to the right pectoral fin, whereas the positive electrode was implanted close to the left pectoral fin. A third electrode (also positive) was implanted in between and approximately 3 cm caudal to the pectoral fins, forming a triangle in relation to the fish’s heart (i.e., Einthoven's triangle). A fourth electrode was submerged in the experimental water tank to ground noise. In addition, a polyethylene cannula (PE-50) was implanted into the peritoneal cavity guided by an 18-gauge needle for pharmacological drug injections. The surgical procedures lasted less than 30 min in total.

### Short-Term Recovery Group

In the short-term recovery group, the ligation or sham surgery, the ECG electrode, and the cannula implantations were all performed during the same surgical procedure. The fish were then allowed to recover for 3 days and held individually in opaque cylindrical plastic tubes (length: 380 mm diameter: 90 mm) submerged in a holding tank (105 L) supplied with aerated 10°C freshwater until experiments started (see *Experimental Protocol*).

### Long-Term Recovery Group

In the long-term recovery group, coronary ligation or sham surgery was performed in all fish at once and the fish were then allowed to recover in their holding tanks for 16 to 24 wk (freshwater at 10°C; 12:12 h photoperiod) while being fed three times a week. After this recovery period, the fish underwent another surgical intervention to implant the ECG electrodes and cannula and were then treated identically as the short-term recovery group (see *Short-Term Recovery Group*). The different length of the postligation period was because the subsequent experimental protocol was only possible to perform on four fish simultaneously. To allow for fish identification (sham vs. ligation) in the long-term recovery group, the fish were marked with colored elastomer (Northwest Marine Technology, Inc., Anacortes, WA) injected subcutaneously in the periorbital region.

### Experimental Protocol: Pharmacological Manipulations of Autonomic Cardiac Regulation

In both short-term and long-term recovery groups, resting heart rate was recorded for at least 1 h on the third day after ECG implantation in unanesthetized fish. After this baseline recording, we initiated the experimental protocol for the blockade of the sympathetic and parasympathetic tone on the heart. To block muscarinic receptors, the fish received an intraperitoneal injection of atropine sulfate salt monohydrate (1.2 mg·kg^−1^, A0257; Sigma-Aldrich), followed by an injection of 0.5 mL of saline to flush the catheter, and recordings of heart rate occurred for an additional 1 h. Subsequently, fish were injected with the β-adrenergic receptor antagonist sotalol hydrochloride (2.7 mg·kg^−1^, S0278; Sigma-Aldrich) followed by a saline injection to achieve a full autonomic blockade, and heart rate was recorded for another 1 h.

### Coronary Artery Vascular Filling

After completion of the experimental protocol in both short- and long-term recovery groups, fish were again anesthetized, and the coronary circulation was examined. For this, sham or ligated fish were positioned on their left lateral side on a surgery table and maintained anesthetized (see *Surgical Procedures*). An incision (∼4 cm) was performed on the lateral side behind the pectoral fin. The celiacomesenteric artery was exposed and dissected free from the surrounding tissue and cannulated with a PE-50 catheter whereafter 0.2 mL of 5,000 IU·mL^−1^ of heparin solution was injected. The fish were then placed ventral side up with the heart exposed and the atrium was cut open allowing blood to be drained. A peristaltic pump was used to infuse a vasodilatory buffer solution (4 mg·L^−1^ of papaverine in phosphate-buffered saline + 50·IU·mL^−1^ of heparin) via the celiacomesenteric artery cannula to clear out blood in the coronary circulation. A yellow-colored Microfil silicone rubber compound (Flow Tech, Inc., Boulder, CO) was then injected through the celiacomesenteric artery. Once the coronaries were completely filled, as indicated by casting fluid appearing in the cut atrium, a 2-0 silk suture was tied around the entire ventral aorta to occlude the main coronary artery preventing backflow of the perfusate. The heart was then covered with gauze soaked with phosphate-buffered saline (PBS) to prevent it from drying and allowed to sit for approximately 30 min for the microfil silicone compound to start curing. Subsequently, the heart was harvested and fixed in 4% paraformaldehyde (4% in PBS) at 4°C for 24 h and later transferred to 70% ethanol.

### Data Acquisition and Calculations

The ECG signals were amplified using a differential amplifier (Bio Amp FE231, ADInstruments, Sydney, Australia) and sampled at a sampling rate of 1 kHz using LabChart software (version 7.3.7, ADInstruments, Sydney, Australia). Two of the bipolar leads were recorded in real time, that is leads I and II, whereas lead III was calculated using the ECG lead setup from LabChart (lead II – lead I). From each ECG recording, we averaged 200 sequential heartbeats that did not contain any obvious ectopic beats or other artifacts, for which we determined QRS complex voltage in millivolts and number of spikes within the QRS complex (fragmented QRS), as well as the PR, QRS, and QT interval in seconds. QT interval was measured from the onset of the QRS complex to the end of the T wave. The PR, QRS, and QT interval were averaged from all three leads. Number of spikes within the QRS complex and the QRS amplitude were summed among all three leads (33). Cholinergic and adrenergic tones were calculated based on the cardiac intervals, i.e., the R to R wave (RR) intervals. The intrinsic heart rate was obtained after the complete autonomic blockade and cholinergic and adrenergic tone were calculated according to the study by Altimiras et al. (34):

(*1*) $$T_{\text{Chol}}=\frac{{\text{RR}}_{\text{cont}}-{\text{ RR}}_{\text{musc}}}{{\text{RR}}_{0}}\;\times 100$$

(*2*) $$T_{\text{Adre}}=\frac{{\text{RR}}_{0}-{\text{ RR}}_{\text{musc}}}{{\text{RR}}_{0}}\;\times 100$$

where *T*_Chol_ is the cholinergic tone (%), *T*_Adre_ is the adrenergic tone (%), RR_cont_ is the RR interval control, RR_musc_ is the RR interval after muscarinic receptor blockade, and RR_0_ is the RR interval after total autonomic block.

### Tissue Preparation and Photographing

All hearts were photographed in the ventrodorsal projection by a Canon EOS 40 D camera (Canon EF 100 mm f/2.8L Macro IS USM Lens) mounted in a lighting unit (Kaiser Fototechnik RB 218N HF, Buchen, Germany) before further preparation for histology. For that, a Petri dish half filled with agar containing a hole in the center, allowing the heart to be positioned in the desired orientation for photographing. The petri dish was filled with ethanol 70% so that the heart could be completely submerged. In addition, the capacity for rapid coronary revascularization was further investigated by using computed tomography (CT) images of a casting compound-perfused heart from one representative trout that was allowed to recover for 10 days after ligation surgery.

### Histological Staining and Immunohistochemistry

Tissue preparation and staining were previously described (8). Briefly, hearts were dehydrated in a graded series of 70%, 80%, 90%, and 99.5% (repeated three times) ethanol, cleared in Histo-Clear solution and embedded in paraffin wax. Paraffin-waxed samples were sectioned at 5-μm thickness and stained for hematoxylin and eosin and picrosirius red staining. For details regarding the Picrosirius staining protocol, see the study by Zena et al. (8). To visualize hypoxic regions in the heart, pimonidazole hydrochloride was dissolved in saline (60 mg·kg^−1^; Hypoxyprobe-1 kit, Hypoxyprobe, Inc., Burlington, MA) and injected intraperitoneally into three fish from the short-term recovery group (1 sham-operated and 2 coronary-ligated) and in eight fish from the long-term recovery group (4 sham-operated and 4 coronary-ligated). Hypoxyprobe was injected 24 h before the fish were euthanized by MS-222 overdose (tricaine methanesulfonate, 150 mg·L^−1^ buffered with NaHCO_3_, 300 mg·L^−1^), at which point the heart was harvested, fixed in 4% PFA, dehydrated, and embedded in paraffin wax. To optimize pimonidazole detection, antigen retrieval was performed on deparaffinized samples using a citrate buffer solution for 10 min at 95°C. After endogenous peroxidase activity was blocked with 3% hydrogen peroxide for 15 min, the primary antibody anti-pimonidazole mouse IgG_1_ monoclonal antibody (Mab1) (dilution 1:50) was added overnight in a humidified chamber at 4°C. Tissue sections were subjected to Vector Laboratory VECTASTAIN Elite ABC-HRP Kit (no. PK-6100) followed by development using the Vector NovaRED peroxidase substrate kit (Vector Laboratories no.SK-4800) both according to manufacturer instructions (35).

### Statistical Analysis

Statistical analyses were performed using R software v. 1.1.383 (http://www.R-project.org/). To assess the effect of surgery (sham vs. ligation) between short-term and long-term recovery groups, we fitted linear models using the “lm” function from the stats package in R on body characteristics (body mass, standard length, and condition factor), resting and intrinsic heart rate, autonomic tone (adrenergic and cholinergic tones), QRS amplitude, QRS interval, fragmented QRS, and QT interval. Because the QT interval is prolonged at slower heart rates and shortened at faster heart rates, heart rate was included as a covariate in the analysis. Moreover, since increases in heart rate induced by exercise or pharmacologically by atropine are associated with a decrease in the PR interval (36), we used a linear model using the “lm” function to evaluate the effect of changes in heart rate by atropine on the PR interval. When an effect of heart rate on QT and PR interval was identified, it was included as a covariate in the model, and all mean values for QT and PR interval were standardized based on an averaged heart rate. For all the above variables, an additional analysis was performed by using linear models to investigate the influence of time since sham/ligation surgery (i.e., from 117 to 173 days) among fish within the long-term recovery group. However, time since surgery had no effect on any of the variables analyzed in the present study. A *t* test was performed between sham and ligated fish within each short-term and long-term recovery group. All values are presented as means ± SE. Statistical significance was accepted at *P* ≤ 0.05. Normality of the residuals was visually inspected by using histograms and boxplots and when necessary, appropriate transformations were performed (log- or square-root transformation).

## RESULTS

### Body Characteristics of Experimental Groups

All fish survived the surgical procedures. Body size metrics are summarized in Table 1. There were no morphometric differences between the sham-operated and coronary-ligated fish in either the short-term or long-term recovery groups. However, the long-term recovery group had larger body mass, standard length, and condition factor compared with the short-term recovery group, indicating that trout grew bigger over time independent of treatment (sham vs. ligated).

### Detection of Hypoxia in the Compact Myocardium following Short-Term Recovery from Coronary Ligation

**Figure 1.**
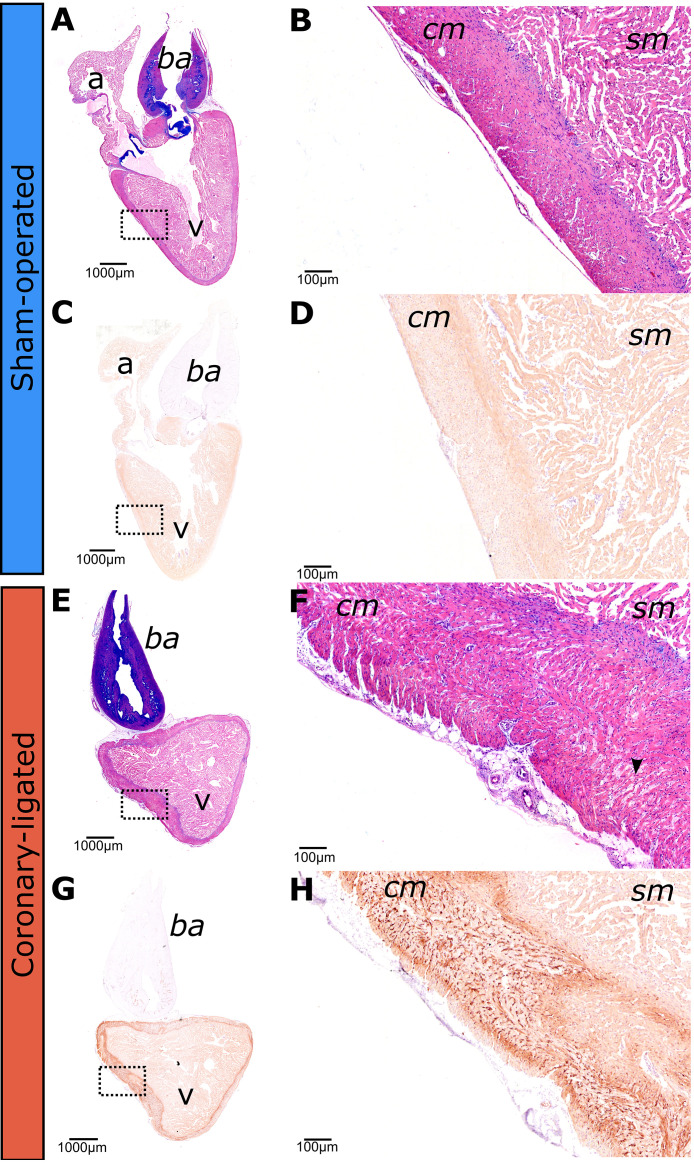
Short-term effects of coronary ligation or sham operation on histopathology of the rainbow trout heart. Whole heart histological sections of the rainbow trout heart in sham-operated (*A*, *B*) and coronary-ligated (*E*, *F*) fish were examined 3 days following sham/ligation surgery. *B* and *F* display magnified images of the boxed regions in *A* and *E*, respectively. Brightfield images stained with hematoxylin and eosin. An arrowhead indicates thin myofiber waviness separated by spaces, representing intercellular edema in coronary-ligated fish. Immunolabeled sections were used to detect Hypoxyprobe (Pimonidazole hydrochloride) staining (in brown) in sham-operated (*C*, *D*) and coronary-ligated (*G*, *H*) rainbow trout hearts. These sections were examined for 3 days following sham/ligation surgery. Notably, the compact myocardium in coronary-ligated fish (*G* and *H*) shows areas with high staining intensity (i.e., hypoxic areas) compared with the sham fish (*C*, *D*). cm, compact myocardium; sm, spongy myocardium; a, atrium; v, ventricle; ba, bulbus arteriosus.

Pimonidazole hydrochloride hypoxyprobe staining revealed normal myocardial tissue morphology and absence of hypoxic areas in the heart of the sham-operated fish (Fig. 1, *A*–*D*), while we detected a robust hypoxyprobe signal in localized areas of the compact myocardium in the coronary-ligated fish from the short-term recovery group (Fig. 1, *E*–*H*). In addition, a detailed histologic examination showed that the compact myocardium from the coronary-ligated fish exhibited thinned and stretched wavy myocardial fibers (Fig. 1*F*).

### Ventricular Myocardial Remodeling and Coronary Artery Revascularization after Long-Term Recovery from Coronary Ligation

**Figure 2.**
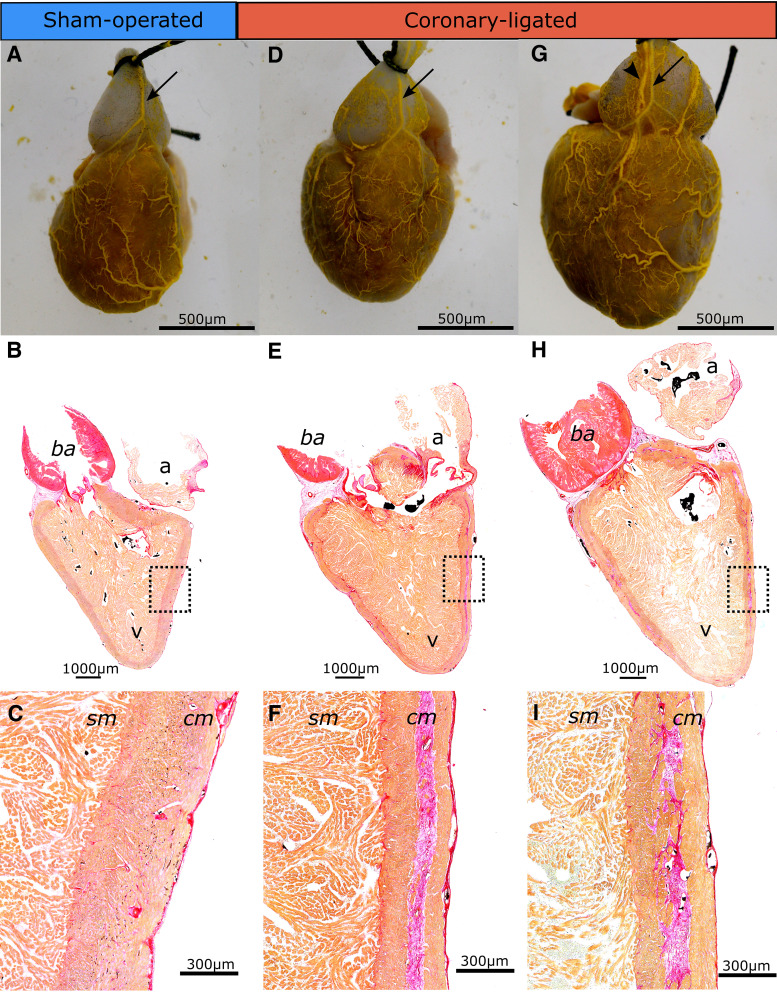
Long-term effects of coronary ligation or sham operation on heart morphology and on histopathology of the rainbow trout heart. Coronary vasculature of one sham-operated (*A*) and two coronary-ligated (*D* and *G*) trout after coronary artery vascular filling with Microfil silicone compound. Note that the coronary vessels depicted in *D* and *G* represent the regrowth that occurred during the recovery period following ligation surgery. Black arrows show the main coronary artery, whereas the arrowhead shows the coronary vein. Whole heart histological sections of one sham-operated (*B*: 131 days) and two coronary-ligated (*E*: 160 days, and *H*: 173 days) rainbow trout hearts. Examples are from the ventricle section stained with picrosirius red that stains collagen in red and muscle tissue in orange. *C*, *F*, and *I* show magnified pictures of the boxed region in *B*, *E*, and *H*, respectively. Note that the compact myocardium in coronary-ligated fish is divided into an outer and inner layer with a layer of collagen in-between. Black spots in the lumen of blood vessels and ventricular lumen are residuals of Microfil injected in the circulation. cm, compact myocardium; sm, spongy myocardium; a, atrium; v, ventricle; ba, bulbus arteriosus.

After long-term recovery, all fish that underwent coronary ligation showed restored coronary supply, as evidenced by a well-developed coronary circulation following vascular filling with the Microfil silicone compound (Fig. 2, *D* and *G*). Histologic evaluation of the same hearts revealed the presence of scar tissue primarily in the median portion of the compact myocardium (Fig. 2, *E*, *F*, *H*, and *I*). In addition, large coronary vessels were observed within the fibrotic tissue, suggesting that the formation of the fibrous midmyocardium layer may occur in parallel with coronary revascularization (Fig. 2*I*). None of the sham-operated fish showed any signs of myocardial fibrosis (Fig. 2, *B* and *C*). Furthermore, the compact myocardium did no longer exhibit any detectable pimonidazole hydrochloride hypoxyprobe staining (Supplemental Fig. S1), which is consistent with a well-revascularized compact myocardium (Fig. 2, *D* and *G*). The capacity for rapid coronary revascularization during myocardial ischemia in juvenile trout can be seen in a fish (representative) that was allowed to recover for 10 days after ligation surgery, demonstrating the onset of revascularization, likely originating from a preexisting mesh-like network of small vessels located on the surface of the bulbus arteriosus (Supplemental Videos S1 and S2). Occasionally, collateral vessels could also be seen coursing alongside the bulbus arteriosus in trout recovered from ligation (Supplemental Video S3).

### Effect of Coronary Ligation on Heart Rate and Autonomic Tones

**Figure 3.**
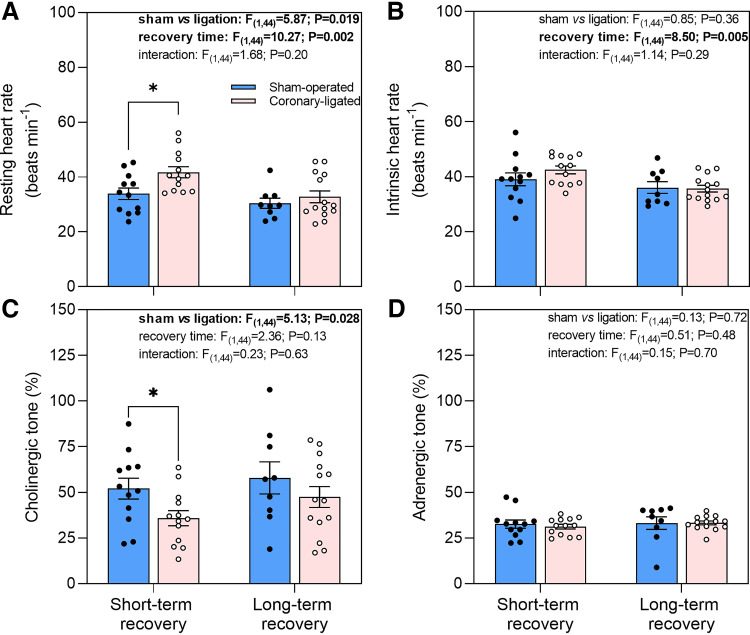
Short- and long-term effects of coronary ligation or sham operation on resting and intrinsic heart rate, and autonomic tone in rainbow trout. Resting heart rate (*A*), intrinsic heart rate (*B*), cholinergic tone (*C*), and adrenergic tone (*D*) in short- and long-term recovered sham-operated and coronary-ligated rainbow trout are presented. Results from the linear model for the respective variables are shown; statistically significant (*P* ≤ 0.05) findings are in bold. *Significant difference between sham operated and coronary ligated trout within short or long-term recovery groups. Data are means ± SE.

Short-term coronary ligation resulted in increased resting heart rate relative to sham-operated fish (41.8 ± 2.0 vs. 33.9 ± 2.1 beats/min; *t*_44_ = 2.655, *P* = 0.01; Fig. 3*A*). However, after full autonomic blockade with atropine and sotalol, there was no significant difference in intrinsic heart rate between groups (39.1 ± 2.3 vs. 42.5 ± 1.4 beats/min, *t*_44_ = 1.403, *P* = 0.17; Fig. 3*B*). The elevated resting heart rate in coronary-ligated fish occurred mainly as a result of lowered cholinergic tone on the heart (35.8 ± 4.1 vs. 52.0 ± 5.7%, *t*_44_ = −1.982, *P* = 0.05; Fig. 3*C*), while adrenergic tone was unaltered (31.2 ± 1.3 vs. 32.6 ± 2.2%; Fig. 3*D*).

In the long-term recovery group, there was no difference between sham-operated and coronary-ligated fish in resting (30.4 ± 1.8 vs. 32.8 ± 2.1 beats/min; Fig. 3*A*) and intrinsic heart rate (36.1 ± 2.1 vs. 35.7 ± 1.2 beats/min; Fig. 3*B*), or in cholinergic (57.9 ± 8.8 vs. 47.4 ± 5.7%; Fig. 3*C*) and adrenergic tone (33.1 ± 3.5 vs. 33.2 ± 1.0%; Fig. 3*D*).

### Effect of Coronary Ligation on Ventricular Depolarization (QRS Characteristics) and Repolarization (QT Interval)

**Figure 4.**
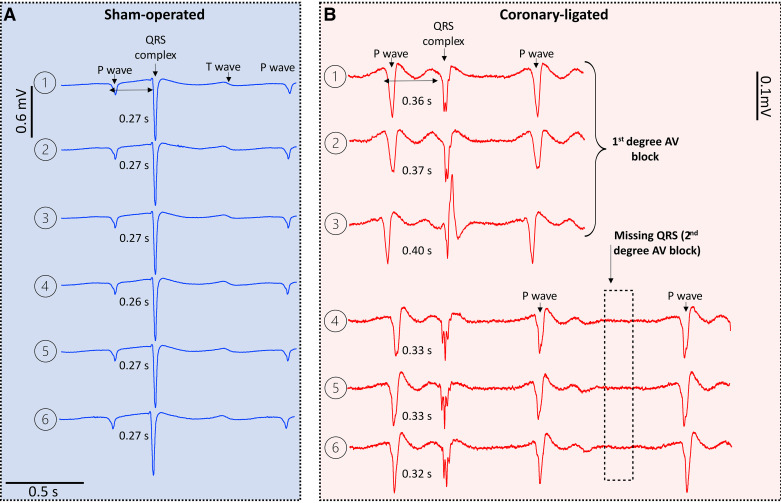
Representative short-term effects of coronary ligation and sham operation on the ECG of rainbow trout. Temporal changes (from 1 to 6) in the ECG of one sham-operated (*A*) and one coronary-ligated (*B*) rainbow trout. ECG recordings in *A* and *B* show the average ECG obtained from 20 consecutive heartbeats (a total of 120 heartbeats) averaged from lead II after atropine injection. Note that in *B*, the PR interval is prolonged (first-degree atrioventricular block) at *point 1* compared with *point 1* in A. The PR interval gradually prolongs (from *1* to *3*) until a nonconducted P wave occurs, that is, a missing QRS (*points 4–6*; dotted rectangle in *B*). A skipped QRS occurs every other beat, leading to a collapse in heart rate. The heart rate in *A* is 54 beats/min, whereas in *B*, the heart rate is 67 beats/min between *points 1* and *3* when a second-degree atrioventricular block occurs, causing the ventricular rate to drop to 33 beats/min, whereas the atrial rate remains at 66 beats/min (2:1 atrioventricular block). The ECG traces in *B* were magnified to facilitate clear visualization of the waves. Note the reduced QRS amplitude (almost equivalent to the size of the P wave) and the presence of spikes within the QRS complex (i.e., fragmented QRS).

**Figure 5.**
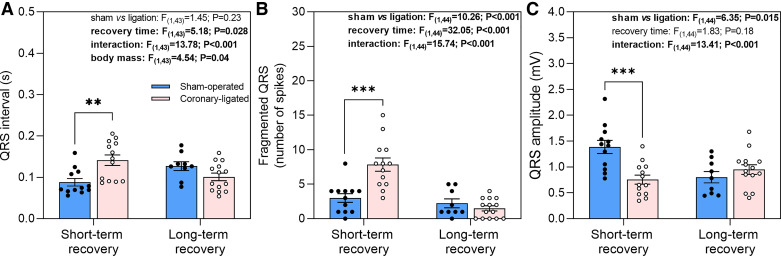
Short- and long-term effects of coronary ligation or sham operation on QRS characteristics of rainbow trout. QRS interval (*A*), fragmented QRS (*B*), and QRS amplitude (*C*) in short- and long-term recovered sham-operated and coronary-ligated rainbow trout are presented. Results from the linear model for the respective variables are shown; statistically significant (*P* ≤ 0.05) findings are in bold. Asterisks denote significant difference between sham-operated and coronary-ligated trout within short or long-term recovery groups. *P < 0.05, **P < 0.01. Data are means ± SE.

Short-term coronary ligation significantly affected the QRS morphology (Figs. 4 and 5). There was a ventricular activation delay as indicated by prolonged QRS interval (ligated: 0.14 ± 0.013 vs. sham: 0.087 ± 0 0.009 s; *t*_43_ = 3.421; *P* = 0.0048; Fig. 5*A*), as well as an inhomogeneous activation of the ventricular myocardium as suggested by the large number of spikes (fragmented QRS) within the QRS (ligated: 7.8 ± 1.0 vs. sham: 3.0 ± 0.6, *t*_44_ = 5.051, *P* < 0.0001; Figs. 4*B* and 5*B*). In addition, coronary-ligated trout exhibited reduced QRS amplitude (ligated: 0.7 ± 0.1 vs. sham: 1.4 ± 0.1 mV; *t*_44_ = −4.343; *P* = 0.0004; Figs. 4*B* and 5*C*).

None of the QRS characteristics were affected by ligation in the long-term recovery group. Thus, between sham-operated and coronary-ligated fish QRS interval (0.10 ± 0.009 vs. 0.13 ± 0.01 s; *t*_43_ = −1.852; *P* = 0.07; Fig. 5*A*), number of spikes within the QRS (1.5 ± 0.4 vs. 2.2 ± 0.6%; *t*_44_ = −0.705; *P* = 0.48; Fig. 5*B*), and QRS amplitude (0.94 ± 0.1 vs.0.80 ± 0.1 mV; *t*_44_ = 0.949; *P* = 0.35; Fig. 5*C*) did not differ, respectively.

**Figure 6.**
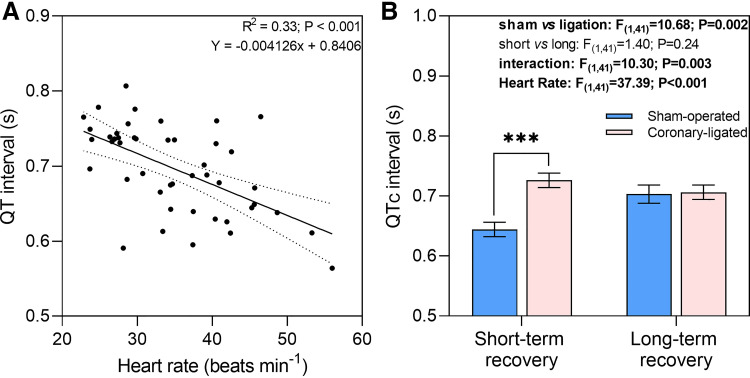
Short- and long-term effects of coronary ligation or sham operation on QT and corrected QT (QTc) interval in rainbow trout (*Oncorhynchus mykiss*). Dependence of the QT interval on heart rate in rainbow trout. *A*: each data point represents average of 200 consecutive heart rate values and QT measurements for a given fish from sham-operated and coronary-ligated treatments within the short- and long-term recovery groups (*Y* = 0.07924*x*+0.5509). *B*: corrected QT interval between sham and coronary-ligated fish for each short and long-term recovery group. For illustrative purposes, all mean values have been standardized, using heart rate as a covariate, to an averaged heart rate (i.e., 34.9 beats/min). For that reason, individual data points are not shown for *B*. ***Significant difference between sham-operated and coronary-ligated trout within the short-term recovery group (*P* < 0.001).

Increases in heart rate caused the QT interval to shorten *(R^2^* = 0.33; *P* < 0.001; Fig. 6*A*). When the QT interval was adjusted based on the fish’s resting heart rate (i.e., corrected QT), coronary-ligated fish from the short-term recovery group exhibited a significantly higher QT interval compared with sham-operated fish (0.13 ± 0.01 vs. 0.10 ± 0.009 s; *t*_41_ = 4.236; *P* < 0.001; Fig. 6*B*). In the long term, however, the QT interval normalized in coronary-ligated fish relative to sham fish (Fig. 6*B*).

### Effect of Coronary Ligation on Atrioventricular Conduction Patterns

**Figure 7.**
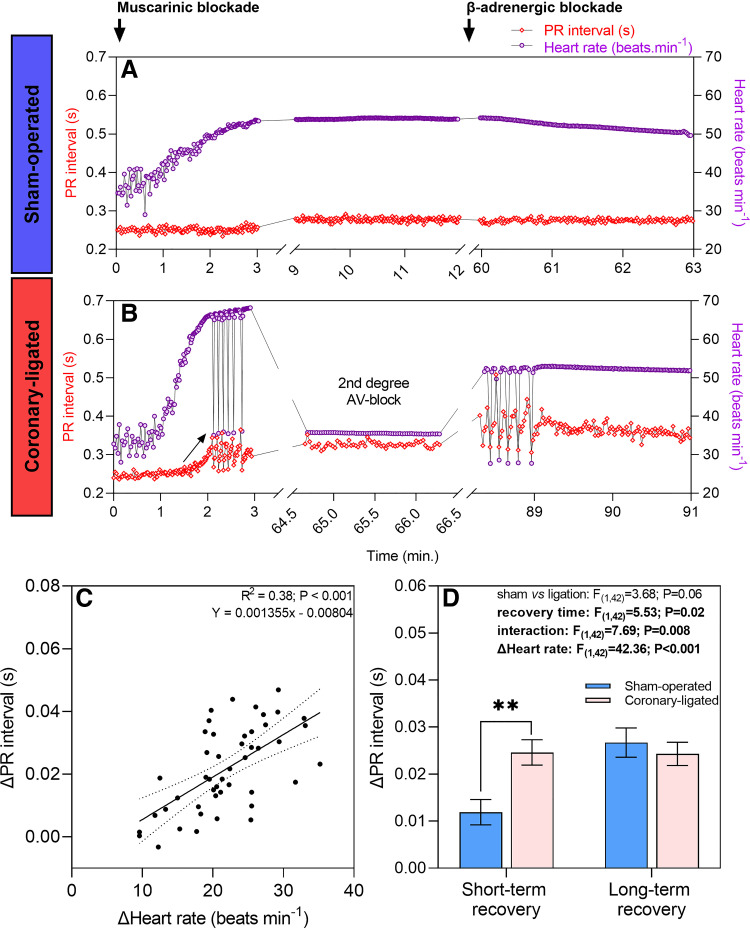
PR interval and heart rate recordings following atropine and sotalol in sham-operated and coronary-ligated rainbow trout (*Oncorhynchus mykiss*). *A* and *B*: show a representative recording from a sham-operated and a coronary-ligated fish, respectively, 3 days after sham/ligation surgery. Heart rate increased after atropine in both fish. Although PR interval remained relatively constant after atropine in the sham fish, it gradually prolonged in the ligated fish (arrow) until it turned into a 2:1 atrioventricular (AV) block, causing a reduction in heart rate. When sotalol was injected, the 2:1 AV block became intermittent until the blockade was alleviated as heart rate was gradually reduced in the coronary-ligated fish. Yet, the PR interval remained prolonged relative to the sham fish. Red diamonds represent the instantaneous PR interval, whereas purple circles represent the heart rate. *C*: dependence of PR interval on changes in heart rate caused by atropine (effect of atropine – baseline). Each data point represents average of 200 consecutive Δheart rate values and ΔPR measurements for a given fish from sham-operated and coronary-ligated treatments within the short and long-term recovery groups (*Y* = 0.001355*x* − 0.00804). *D*: corrected PR interval between sham and coronary-ligated fish for each short- and long-term recovery group after atropine. For illustrative purposes, all mean values have been standardized, using changes in heart rate as a covariate, to an averaged heart rate (i.e., 21.7 beats/min). For that reason, individual data points are not shown for *D*. **Significant difference between sham-operated and coronary-ligated trout within the short-term recovery group (*P* < 0.01).

After atropine treatment, some coronary-ligated fish in the short-term recovery group (3 of 13 fish) developed a second-degree atrioventricular block as indicated by a fixed ratio of 2 P waves for each QRS complex (referred to as a 2:1 conduction ratio) instead of the usual 1:1 relationship (Figs. 4 and 7*B* and Supplemental Fig. S2). Furthermore, a significant positive effect of heart rate on the PR interval was observed after the treatment with atropine [*F*(1,42) = 42.36; *P* < 0.001; Fig. 7, *C* and *D*]. When changes in PR interval were adjusted based on the effect of changes in heart rate, coronary-ligated fish from the short-term recovery group exhibited a significantly higher change in PR interval compared with sham-operated fish (0.025 ± 0.003 vs. 0.012 ± 0.003 s; *t*_42_ = 3.293; *P* = 0.002; Fig. 7*D*). In the long term; however, the PR interval normalized in coronary-ligated fish relative to sham fish. The three coronary-ligated fish that manifested second-degree atrioventricular block after atropine administration restored a normal P:QRS ratio (1:1 ratio) after sotalol treatment (Fig. 7*B* and Supplemental Fig. S2). No second-degree atrioventricular block was observed in any of the fish from the corresponding sham group (Fig. 7) nor in the fish from the long-term recovery group, regardless of whether they were sham-operated or coronary-ligated.

Changes in heart rate achieved pharmacologically by intraperitoneal atropine injection (present data; Supplemental Fig. S3, *A*, *E*, and *F*) or by using a chasing stress protocol for a maximum of 5 min or until fatigue to elicit a maximum cardiorespiratory response (Supplemental Fig. S3, *C*, *G*, and *H*; representative data reassessed from Ref. 8) show that sham-operated trout from both treatment groups displayed minimal changes in the PR interval despite increases in heart rate. On the other hand, coronary-ligated trout in the short-term recovery group exhibited an exponential rise in PR intervals as the heart rate peaked at approximately 65 beats/min (Supplemental Fig. S3, *B* and *D*). Subsequently, the heart rate dropped to resting values due to the emergence of second-degree atrioventricular blocks (Supplemental Fig. S3, *B* and *D*).

## DISCUSSION

### Short-Term Recovery from Coronary Ligation

#### Gross morphology, histopathology, and cardiac remodeling.

The gross morphology examination revealed a notable pale discoloration in the heart subjected to coronary ligation. Upon further histopathological analysis, we observed thin wavy myocytes usually separated by open spaces (8), suggestive of intercellular edema. Wavy myocytes are a common finding in human heart infarcts, which results from the excessive stretching of noncontractile fibers during bulging of the ischemic tissue that occurs during systole (37). This suggests that myocardial fiber waviness can be an important marker for recent cardiac ischemic injury also in salmonids. This finding could also serve as a differential diagnosis from infectious diseases affecting the heart such as cardiomyopathy syndrome, heart and skeletal muscle inflammation, and pancreas disease, all of which induce notable heart inflammation (38), akin to the inflammatory response in rainbow trout hearts subjected to acute coronary artery occlusion (8, 24).

Consistent with studies on acute (within 7 min) coronary ligation in rainbow trout (20, 21, 24), we found; atrioventricular conduction delay (e.g., first- and second-degree atrioventricular blocks), depolarization abnormalities such as QRS interval prolongation, and QRS amplitude reduction (i.e., low QRS voltage) 3 days after ligation in the short-term recovery group. This suggests these ECG changes persist for at least a few days from the onset of ischemia. Furthermore, we found two additional biomarkers of cardiac ischemia, such as fragmented QRS morphology and prolonged QT interval in nonanesthetized trout that were not previously found in anesthetized fish (24). 

#### Atrioventricular conduction time.

Coronary ligation caused a significant prolongation of the PR interval, resembling a typical mammalian first-degree atrioventricular block, but this only occurred in trout with elevated heart rates after atropine treatment. This slowing of the PR interval is probably a result of delayed atrioventricular conduction through the atrioventricular canal, which is formed by a ring of compact myocardium (39, 40), and that was presumably rendered ischemic in coronary-ligated trout. Moreover, in 23% of all coronary-ligated trout, this first-degree AV block developed into a second-degree AV block, which caused intermittent failure of AV conduction resulting in missing QRS complexes. The block cycle occurred at a fixed ratio of 2 P waves for each QRS complex (i.e., a 2:1 conduction ratio; also known as “advanced heart block”) (41, 42). This caused the heart rate to decrease roughly to preatropine values. It is noteworthy that the observed 2:1 second-degree AV block was characterized by a progressive PR interval prolongation in the cardiac cycles preceding the cycle displaying a nonconducted P wave. This is similar to the classic Mobitz type I second-degree AV block (also known as Wenckebach block) found in humans (43). The administration of the β-adrenergic blocker sotalol led to a decrease in the sinus rate and restored 1:1 AV conduction in all trout that initially developed a 2:1 block following atropine treatment. This emphasizes that AV conduction abnormalities in trout during myocardial ischemia are strongly influenced by the fish's heart rate. This also corroborates our previous results on anesthetized trout, showing that bradycardia improves AV conduction time and abolishes 2:1 block during acute myocardial ischemia (24).

In mammals, exercise leads to increased heart rate and shortening of the AV node conduction time, and therefore generally a shorter PR interval at high heart rates (36, 44, 45). This occurs because the mammalian AV node is innervated by the autonomic nervous system and is mediated by a withdrawal of parasympathetic (vagal) tone (44). Similarly, the PR interval gradually shortens as the heart rate increases with progressive acute warming in rainbow trout (46). Since both limbs of the autonomic nervous system innervate the atrioventricular canal in the fish heart (47), it seems likely that atrioventricular conduction time would also shorten at high heart rates following swimming activity in trout. Therefore, we reassessed ECG data from a previous study on one sham and one coronary-ligated trout before and after a chase stress protocol (8). Interestingly the tachycardia following a chase stress protocol did not induce apparent changes in the PR interval, at least not in the examined fish. However, it is crucial to emphasize that the absence of observed changes does not suggest that the PR interval is resistant to shortening during increased heart rates. A gradual reduction in the PR interval was demonstrated as the heart rate increased due to warming in rainbow trout (46). However, our analysis of the effect of atropine, increasing the trout’s heart rate, showed a positive relationship with the PR interval. This means that atropine-induced increases in heart rate led to a prolongation of the PR interval. Nevertheless, after heart rate correction, a prolonged PR interval became evident only in the ligated fish from the short-term recovery group, meaning impaired AV conduction (i.e., first-degree AV block) during myocardial ischemia.

#### QRS characteristics.

In addition to delayed ventricular electrical activation, coronary-ligated fish showed reduced QRS voltage amplitude. Reduced QRS amplitude in humans is typically due to intrinsic myocardial problems such as myocardial infarction and myocarditis and indicates extensive myocardial injury with loss of viable myocardium mass (48, 49). The current finding is also consistent with the effects of acute coronary ligation previously observed in anesthetized rainbow trout (20, 21, 24).

Prolonged QRS waves and reduced amplitude are markers found within minutes (∼7 min) from the onset of ischemia in anesthetized trout (24). The present data show that these abnormalities persist at least 3 days from the start of an ischemic insult. Another QRS abnormality found within 3 days after coronary ligation in the present study was fragmentation of the QRS complex. This indicates inhomogeneous ventricular activation combined with myocardial conduction delays, which occur due to the shifting of the depolarization wave within and around areas affected by myocardial ischemia or necrosis (50). Therefore, QRS fragmentation associated with myocardial ischemia in rainbow trout could serve as a potential supplementary marker for predicting mortality risk in salmonid fish.

#### QT interval: ventricular depolarization and repolarization duration.

Measures of the QT interval are used to assess the risk of malignant arrhythmias and sudden death associated with a prolonged QT interval in humans. However, as increases or decreases in heart rate normally shorten or lengthen the QT interval, respectively, it is crucial to correct this measurement for heart rate changes (51). Thus, it is not surprising that we identified a negative relationship between the uncorrected QT interval and heart rate across treatment groups. However, after heart rate correction, a prolonged QT interval became evident only in the ligated fish. In humans, a prolonged QT interval underlies specific ventricular arrhythmias like Torsades de pointes, a polymorphic ventricular tachycardia characterized on the electrocardiogram as oscillatory changes in the QRS amplitude around the isoelectric line, which can degenerate into ventricular fibrillation and sudden death (52, 53). Even so, despite the prolonged QT interval in coronary-ligated trout, we did not observe any ECG alterations suggestive of such arrhythmias. Although additional studies are necessary to fully understand the impact of a prolonged QT interval on fish cardiac function, our findings suggest that QT interval may be used as an early indicator of acute myocardial ischemia in fish.

### Long-Term Recovery from Coronary Ligation

#### Gross morphology, histopathology, and cardiac remodeling.

There were no mortalities following surgery in both long-term recovery groups, and coronary ligation resulted in the development of ventricular fibrotic scar tissue. The transmural ischemia following coronary ligation (as evidenced by a strong hypoxyprobe staining observed throughout most of the compact myocardium) resulted in the formation of scar tissue over the subsequent long-term healing process, which was primarily confined to the mid-portion of the compact myocardium while the surrounding myocardium appeared healthy and showed no signs of ischemia. Similar cardiac remodeling as a consequence of myocardial ischemia was previously described in juvenile trout examined after a shorter (from 33 to 62 days) coronary ligation recovery time (8). This may suggest that the mid-myocardium fibrous tissue could be permanent. Yet, the underlying reasons for this unusual extracellular matrix deposition following complete compact myocardial ischemia in trout remains unknown.

We have previously suggested that the midmyocardial fibrous layer in rainbow trout demarked the inner region of the compact myocardium that continued to receive oxygen from the luminal venous blood, while the outer compact layer would be supplied by newly formed coronary vessels (8). Indeed, all coronary-ligated fish in the long-term recovery group showed a functional coronary vasculature and no signs of compact myocardium ischemia, confirming that new coronary vessels had formed during the recovery period. In fact, newly formed coronary vessels appear as early as 10 days after coronary artery ligation, and it appears they originate from a pre-existing mesh-like network of small vessels on the bulbus arteriosus (Supplemental Videos S1 and S2). This network appears to be independently perfused from the main coronary vessels, as some ligated fish from the short-term recovery group displayed patent vessels when infused with Microfil silicone compound even if the main coronary artery was occluded. It remains unclear whether the blood supply to the mesh-like network originates independently from the hypobranchial artery, which branches to form the main coronary artery (22), or if it arises from a branch of the hypobranchial artery upstream from the ligation point.

Numerous open large coronary vessels (confirmed with Microfil injections) were observed within the midmyocardial fibrous layer. This suggests that restoration of blood supply to compact myocardium in rainbow trout may underlie tissue recovery, which may encompass processes such as regeneration thereby explaining the presence of viable and functional (nonischemic) neighboring myocardium in the long term. These processes are vital for promoting healing, preventing further damage, and ultimately improving overall survival in trout (54). However, in humans, when a major epicardial coronary artery courses within the myocardium, a congenital condition known as myocardial bridging, the “tunneled” coronary artery segment can experience systolic compression due to the overlying myocardial tissue, which may lead to myocardial ischemia, infarction, and even sudden death (55). The presence of midmyocardium layer of loose connective tissue associated with large coronary vessels in the trout heart may suggest an adaptive long-term cardiac remodeling response to myocardial ischemia. This layer could act as a cushion, limiting the compressive forces on the bridged coronary segment during systole (56, 57), especially when fish is operating at high heart rates. Tachycardia can provoke an ischemic effect in the setting of a myocardial bridge due to the alteration of flow dynamics, reducing the time for diastole and increasing reliance on systolic coronary flow for perfusion, which is impaired by the bridge (58).

#### ECG characteristics.

Myocardial scarring underlies the majority of ventricular arrhythmias found in humans (5). Activation in the healed infarcted region is characterized by low-amplitude voltage with localized delays and fractionated areas that involve tortuous or “zig-zag” conduction in and around infarcted regions through isolated bundles of surviving myocytes (59, 60). Despite the presence of significant scar tissue in the hearts of rainbow trout, the electrocardiographic abnormalities detected during the initial stage of ischemia were absent in fish following prolonged recovery from ischemia. Noteworthy, even though there was no difference in QRS amplitude between sham-operated and coronary-ligated fish in the long-term recovery group, the overall amplitude was lower in these larger fish when compared with the smaller fish in the short-term recovery group. Extracardiac factors such as the passive body volume conductor surrounding the heart likely explain the reduced QRS amplitude in the bigger fish (61).

Although QRS amplitude depression serves as a reliable indicator of myocardial ischemia in trout (24), it is important to acknowledge limitations like extracardiac factors that can influence QRS amplitude in diagnostic and management situations in salmonid aquaculture settings. In fact, when collectively analyzing fish from both sham-operated groups, a negative relationship was found between fish body mass and QRS amplitude (data not shown). In addition, considering that farmed salmonids often display notable fat deposits in the epicardium (62) and that greater amounts of cardiac adipose tissue are associated with lower QRS voltages (63), this factor should be taken into account when performing ECG comparisons among farmed salmonids of varying sizes, as well as with wild salmonids.

### Perspectives and Significance

In conclusion, the present study makes a substantial contribution to our comprehension of cardiac remodeling and repair processes in teleost fish, along with shedding light on the significance of electrocardiography in evaluating heart conditions like myocardial ischemia in salmonid fish. Notable ECG changes resembling myocardial ischemia-like ECG in humans such as atrioventricular blocks and abnormal ventricular depolarization and repolarization patterns were found during the acute phase of myocardial ischemia. Yet, the high (100%) survival rates in juvenile rainbow trout after coronary ligation are likely owed to coronary artery regrowth contributing to restored and sustained cardiac function. Unlike the complete fibrotic resolution observed after cardiac injury in other teleost fishes like zebrafish, rainbow trout appear to retain long-lasting scar tissue following myocardial ischemia. This scarring could potentially give rise to distinct electrophysiological characteristics from the surrounding healthy myocardium and create areas of functional heterogeneity that are prone to arrhythmias; however, ECG abnormalities were absent in surviving trout from the long-term recovery group. Therefore, the persistent presence of fibrous tissue, always in association with major coronary vessels, may function as a perivascular cushion of connective tissue, serving to protect the vessel from excessive systolic compression caused by the surrounding compact myocardial tissue. Our discovery reveals the complexity of cardiac restoration in fish and confirms that cardiac repair differs significantly among fish species. These findings not only open new opportunities for comparative studies within a framework of cardiovascular physiology and organ repair but also hold the promise of offering profound insights into the repercussions of heart diseases on the overall health of salmonid fish. In a broader context, this study provides new insights into the evolution and ontogeny of vertebrate cardiac repair and functional restoration.

## DATA AVAILABILITY

Data will be made available upon reasonable request.

## SUPPLEMENTAL DATA

10.6084/m9.figshare.25250902 Supplemental Figs. S1–S3: https://doi.org/10.6084/m9.figshare.25250902.

10.6084/m9.figshare.24948792 Supplemental Videos S1–S3: https://doi.org/10.6084/m9.figshare.24948792.

## GRANTS

This work was supported by Helge Ax:son Johnsons Stiftelse (F22-0130 and F23-0236 to L.A.Z.), the Wilhelm and Martina Lundgren Foundation (2023-GU-4311 to L.A.Z.), The Swedish Research Council for Environment, Agricultural Sciences and Spatial Planning (Svenska Forskningsrådet Formas; 2019-00299 to E.S.), and NordForsk (DigiHeart project 103385 to I.B.J., A.G., and E.S.).

## DISCLOSURES

No conflicts of interest, financial or otherwise, are declared by the authors.

## AUTHOR CONTRIBUTIONS

L.A.Z., A.T.E., M.A., H.S., I.B.J., A.G., and E.S. conceived and designed research; L.A.Z., A.T.E., D.M., and T.M. performed experiments; L.A.Z., T.M., A.P., and A.G. analyzed data; L.A.Z., A.T.E., D.M., T.M., M.A., H.S., A.P., I.B.J., A.G., and E.S. interpreted results of experiments; L.A.Z. and A.P. prepared figures; L.A.Z. and E.S. drafted manuscript; L.A.Z., A.T.E., D.M., T.M., M.A., H.S., A.P., I.B.J., A.G., and E.S. edited and revised manuscript; L.A.Z., A.T.E., D.M., T.M., M.A., H.S., A.P., I.B.J., A.G., and E.S. approved final version of manuscript.
